# Gut microbiome-derived butyrate inhibits the immunosuppressive factors PD-L1 and IL-10 in tumor-associated macrophages in gastric cancer

**DOI:** 10.1080/19490976.2023.2300846

**Published:** 2024-01-10

**Authors:** Seung Yoon Lee, JooYeon Jhun, Jin Seok Woo, Kun Hee Lee, Sun-Hee Hwang, Jeonghyeon Moon, Goeun Park, Sun Shim Choi, So Jung Kim, Yoon Ju Jung, Kyo Young Song, Mi-La Cho

**Affiliations:** a Rheumatism Research Center, Catholic Research Institute of Medical Science, The Catholic University of Korea, Seoul, Korea; b Lab of Translational ImmunoMedicine, Catholic Research Institute of Medical Science, College of Medicine, The Catholic University of Korea, Seoul, Korea; c Department of Biomedicine & Health Sciences, College of Medicine, The Catholic University of Korea, Seoul, Korea; d Departments of Immunobiology and Neurology, Yale School of Medicine, New Haven, CT, USA; e Division of Biomedical Convergence, College of Biomedical Science, Institute of Bioscience & Biotechnology, Kangwon National University, Chuncheon, Korea; f Division of Gastrointestinal Surgery, Department of Surgery, Seoul St. Mary’s Hospital, College of Medicine, The Catholic University of Korea, Seoul, Korea; g Division of Gastrointestinal Surgery, Department of Surgery, Yeouido St. Mary’s Hospital, Seoul, Korea; h Department of Medical Life Sciences, College of Medicine, The Catholic University of Korea, Seoul, Korea

**Keywords:** Gastric cancer, PD-L1, IL-10, microbiome, butyrate, avatar model, *Faecalibacterium prausnitzii*

## Abstract

Early detection and surgical treatment are essential to achieve a good outcome in gastric cancer (GC). Stage IV and recurrent GC have a poor prognosis. Therefore, new treatments for GC are needed. We investigated the intestinal microbiome of GC patients and attempted to reverse the immunosuppression of the immune and cancer cells of GC patients through the modulation of microbiome metabolites. We evaluated the levels of programmed death-ligand 1 (PD-L1) and interleukin (IL)-10 in the peripheral blood immunocytes of GC patients. Cancer tissues were obtained from patients who underwent surgical resection of GC, and stained sections of cancer tissues were visualized via confocal microscopy. The intestinal microbiome was analyzed using stool samples of healthy individuals and GC patients. Patient-derived avatar model was developed by injecting peripheral blood mononuclear cells (PBMCs) from advanced GC (AGC) patients into NSG mice, followed by injection of AGS cells. PD-L1 and IL-10 had higher expression levels in immune cells of GC patients than in those of healthy controls. The levels of immunosuppressive factors were increased in the immune and tumor cells of tumor tissues of GC patients. The abundances of *Faecalibacterium* and *Bifidobacterium* in the intestinal flora were lower in GC patients than in healthy individuals. Butyrate, a representative microbiome metabolite, suppressed the expression levels of PD-L1 and IL-10 in immune cells. In addition, the PBMCs of AGC patients showed increased levels of immunosuppressive factors in the avatar mouse model. Butyrate inhibited tumor growth in mice. Restoration of the intestinal microbiome and its metabolic functions inhibit tumor growth and reverse the immunosuppression due to increased PD-L1 and IL-10 levels in PBMCs and tumor cells of GC patients.

## Introduction

In 2020, gastric cancer (GC) was the fourth-leading cause of cancer death worldwide after lung, colorectal, and liver cancers.^1^ Early detection and surgical treatment are necessary to achieve good outcomes in GC. Stage IV and recurrent GC have a poor prognosis. Therefore, new treatments are needed for GC.^2,3^ Immune checkpoint inhibitors, such as anti-programmed death (PD)-1 or anti-PD-ligand 1 (L1), are used to treat stage IV GC, although the predictors of treatment response are unknown.^4,5^ Because of a lack of survival benefit with current treatments, several ongoing studies are evaluating combination treatments for GC. Tumor-associated macrophages (TAMs), which promote cancer cell metastasis and multiplication, are found in several cancer types.^6^ PD-L1, a target of cancer immunotherapy, is highly expressed in TAMs, which suppresses or kills T cells that prevent cancer progression.^6,7^ The microbiome plays an important role in the functions of immune cells.^8,9^

The microbiome differs between healthy individuals and cancer patients, and may be an indicator of the treatment response to anticancer therapy.^10^ *Helicobacter pylori* is the most common flora in GC, although the underlying mechanism is unclear. Imbalance between the gastric microbiome and host promotes the development of GC.^11^ A recent study showed that the microbiome of GC patients affects the treatment response to immune checkpoint blockade drugs, such as anti-PD-1 and anti-CTLA-4. Increased abundances of *Faecalibacterium* and *Ruminococcacea* are associated with a good treatment response, whereas increased abundance of *Bacteroidetes* is associated with a poor response.^12^

Short-chain fatty acids (SCFAs), including butyrate, acetate, and propionate,^13^ are microbial metabolites that mediate the communication between the intestinal microbiome and the immune system.^14^ Among them, butyrate has the most biological functions. It inhibits histone deacetylase and promotes gene activity through chromatin acetylation.^15–17^ The SCFA levels are reduced in cancer patients and is considered for cancer treatment. It has been reported that acetate inhibited colon cancer cell growth in vitro.^18^ An increasing acetate level has been shown to benefit some cancer types.^19^ In addition, it has been reported anti-tumor effect of propionate. Propionate suppressed proliferation of colon cancer^20^ and induced apoptosis in lung cancer^21^ *in vitro*. *In vivo*, it has been shown the anti-tumor effect of propionate in breast cancer mouse model.^22^ It has been shown that butyrate also has anti-tumor effect in the colon cancer.^23^ However, it has not been reported the association between SCFA, especially butyrate, and the expression of immunosuppressive markers.

In this study, we investigated the associations between the expression levels of PD-L1 and IL-10 in the peripheral blood mononuclear cells (PBMCs) and cancer tissues of GC patients. For the first time, we identified the microbiome metabolites that affect PD-L1 expression in the PBMCs of GC patients and evaluated their *in vitro* effects and anticancer effects on a GC avatar mouse model.

## Materials and methods

### Study population

We enrolled 40 GC patients diagnosed with adenocarcinoma on preoperative endoscopic biopsy. Peripheral blood and cancer tissue samples were collected. The preoperative blood parameters were compared between early GC (EGC) and advanced GC (AGC) groups. Furthermore, fecal samples were collected preoperatively from 39 patients for microbiome analysis. We also enrolled 20 healthy controls (HCs). The pathological stage of GC was classified according to the eighth American Joint Committee on Cancer criteria. The study protocol was approved by the Institutional Review Board of the College of Medicine, Catholic University of Korea (IRB No. KC20TISI0985). Patient records were anonymized and de-identified before analysis.

### Isolation of human PBMCs

Blood samples were obtaining from St. Mary’s Hospital, The Catholic University of Korea, Seoul, Korea. The study participants provided written informed consent. PBMCs obtained from HCs were separated from buffy coats using Ficoll-Hypaque (Pharmacia Biotech, Piscataway, NJ, USA) and red blood cells were removed.

### Fecal DNA extraction, polymerase chain reaction (PCR) amplification, and DNA sequencing

Fecal samples were collected in plastic containers, stored on ice, transported to the research site, and then stored at −70°C within 12 h of arrival. Total DNA was extracted using FastDNA® SPIN Kit for Soil (MP Biomedicals, Solon, OH, USA) in accordance with the manufacturer’s instructions. PCR amplification of the extracted DNA was performed using fusion primers that target the V3–V4 regions of the 16S rRNA gene. For amplification of V3–V4 regions, the 16S sequence was amplified using the forward primers 341 F (5′-AATGATACGGC GACCACCGAGATCTACAC-XXXXXXXX- TCGTCGGCAGCGTC-AGATGTGTATAAGAG ACAG-CCTACGGGNGGCWGCAG-3′; underlined sequence indicates the target region primer) and 805 R (5′-CAAGCAGAAGA CGGCATACGAGAT-XXXXXXXX-GTCTCGTG GGCTCGG-AGATGTGTATAAGAGACAG-GA CTACHVGGGTATCTAATCC-3′). The fusion primers were constructed in the following sequence: P5 (P7) graft binding, i5 (i7) index, NextEra consensus, sequencing adaptor, and target region sequence. DNA amplifications were performed under the following conditions: initial denaturation at 95°C for 3 min, followed by 25 cycles of denaturation at 95°C for 30 s, primer annealing at 55°C for 30 s, extension at 72°C for 30 s, and final elongation at 72°C for 5 min. The PCR product was confirmed using 1% agarose gel electrophoresis and visualized using Gel Doc system (BioRad, Hercules, CA, USA). The amplified products were purified using CleanPCR (CleanNA, Waddinxveen, the Netherlands). Equal concentrations of purified products were pooled, and short fragments (nontarget products) were removed using CleanPCR (CleanNA). The product quality and size were assessed using Bioanalyzer 2100 (Agilent, Palo Alto, CA, USA) and DNA 7500 chip. Mixed amplicons were pooled, and sequencing was performed at Chunlab, Inc. (Seoul, Korea), using MiSeq Sequencing System (Illumina, San Diego, CA, USA) according to the manufacturer’s instructions. Raw 16S rRNA sequences were subjected to bioinformatics analysis using QIIME 2 (version 2019.4),^24^ as described previously.^25^

### Flow cytometry

For intracellular staining, cells were stimulated with 25 ng/mL phorbol 12-myristate 13-acetate and 250 ng/mL ionomycin (both from Sigma-Aldrich, St. Louis, MO, USA) for 4 h in the presence of Golgi-Stop (BD Biosciences, San Diego, CA, USA). PBMCs were stained with surface anti-human CD11c (BV510; BD Biosciences; 563026) and anti-human PD-L1 (FITC; Biolegend San Diego, USA; 393606) antibodies. The cells were permeabilized and fixed with CytoPerm/CytoFix (BD Biosciences) in accordance with the manufacturer’s instructions. After fixation and permeabilization, cells were stained with anti-human CD68 (PE; Biolegend; 12–0689–42) and anti-human IL-10 (APC; Biolegend; 506807). Flow cytometry was performed using CytoFLEX (Beckman Coulter, Fullerton, CA, USA).

### Cell proliferation and CCK8 assays

Cell proliferation was evaluated using cell counting kit-8 (CCK8) assay (Dojindo Laboratories, Kumamoto, Japan) according to the manufacturer’s instructions. AGS GC cells were used to analyze the concentration of sodium butyrate over time. AGS cells were seeded at a concentration of 2 × 10^3^ cells/well in 96-well plates and incubated at 37°C with 5% CO_2_ for 2 days. Then, 10 μL CCK8 assay reagent was added to each well and incubated at 37°C for 4 h. The absorbance at 450 nm was measured every hour using Multiskan Sky Touch (Thermo Fisher Scientific, Waltham, MA, USA).

### Confocal microscopy of immunostained sections

Cancer tissues were collected from patients who underwent surgical resection for GC. Cancer mucosa tissues were snap-frozen in liquid nitrogen and stored at −70°C. Cryosections of cancer mucosa (7 µm thick) were fixed with methanol and acetone, and stained with fluorescein isothiocyanate, anti-human CD68 (PE; Biolegend; 12–0689–42; 1:100), anti-human IL-10 (APC; Biolegend; 506807; 1:100), and anti-human PD-L1 (FITC; Biolegend; 393606; 1:100). After overnight incubation at 4°C and staining, the sections were analyzed using the LSM 510 Meta confocal microscopy system (Carl Zeiss, Oberkochen, Germany). The stained cells were counted at a high magnification by four investigators.

### Immunohistochemistry

Tumor tissues were fixed and embedded in paraffin. Then the tissues were cut into 7 µm thick sections, dewaxed using xylene, and dehydrated using a graded alcohol series. Immunohistochemistry was performed using Vectastain ABC kit (Vector Laboratories, Burlingame, CA, USA). The sections were incubated with anti-PD-L1 (PA5–20343; Invitrogen, Carlsbad, CA, USA), anti-IL-10 (BS-20373 R; Bioss Antibodies Inc., Woburn, MA, USA), and anti-NF-kB (ab16502; Abcam, Cambridge, MA, USA) overnight at 4°C. Then they were incubated with a biotinylated secondary antibody.

### Humanized mouse model for GC treatment

Mice were maintained under specific-pathogen-free conditions and fed standard mouse chow (Ralston Purina, St. Louis, MO, USA) and water ad libitum. The experimental protocol was approved by the Institutional Animal Care and Use Committee of the School of Medicine and the Animal Research Ethics Committee of the Catholic University of Korea (CUCM-2021–0333–05). The study was conducted in accordance with the Laboratory Animals Welfare Act, Guide for the Care and Use of Laboratory Animals. We used 6- to 8-week-old female NOD/scid/IL-2 Rγ^–/–^ mice (NOD.Cg-*Prkdc*^scid^*IL2rg*^tm1Wjl^/SzJ; hereafter, NSG) obtained from The Jackson Laboratory (Bar Harbor, ME, USA). The mice were maintained under specific-pathogen-free conditions in an animal facility and fed autoclaved food and water. To develop the humanized model, freshly isolated PBMCs from GC patients were injected intraperitoneally into NSG mice (5 × 10^6^/mice). After 1 week, 5 × 10^6^ AGS cells were injected subcutaneously into the flank of mice. The surrounding flank area was shaved before inoculation. Tumor volume was measured three times a week as 0.52 × (short axis)^2^ × (long axis) based on the volume formula for spheres (4/3 πr.^3^ When tumor volume reached 100–300 mm^3^, 200 mg/kg/day sodium butyrate was administered orally. Vehicle-treated animals were administered an equivalent quantity of saline.

### Enzyme-linked immunosorbent assay (ELISA)

IL-10 levels in culture supernatants of human PBMCs were measured using sandwich ELISA (DY217b; R&D system, Minneapolis, MN, USA). Horseradish peroxidase-avidin (R&D Systems) was used for color development. Supernatant samples were stored at −20°C until use. The absorbance at 405 nM was determined using Multiskan Sky Touch (Thermo Fisher Scientific).

## Statistical analysis

Statistical analyses were performed using GraphPad Prism (version 8.01; GraphPad Software Inc., San Diego, CA, USA). One-way analysis of variance was used to evaluate differences among more than two groups. Bonferroni *post hoc* test was used to analyze significant differences among groups. Numerical data were compared between two groups using Mann-Whitney U test or unpaired Student’s t-test. Data are presented as mean ± standard error of the mean. P-value <0.05 was considered indicative of statistical significance.

## Results

### Increased levels of immunosuppressive markers in dendritic cells and macrophages of AGC patients

**Figure 1.**
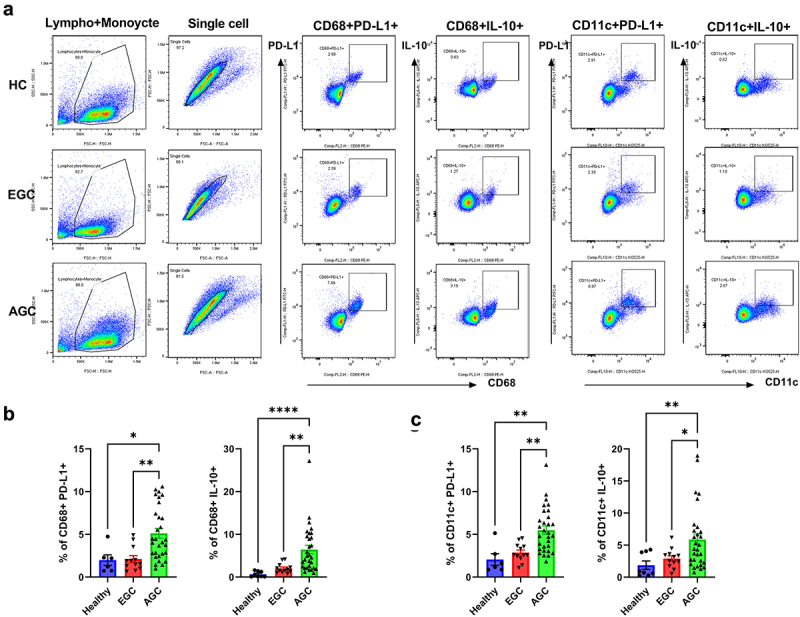
Increased levels of immunosuppressive markers in the PBMCs of AGC patients.

**Figure 2.**
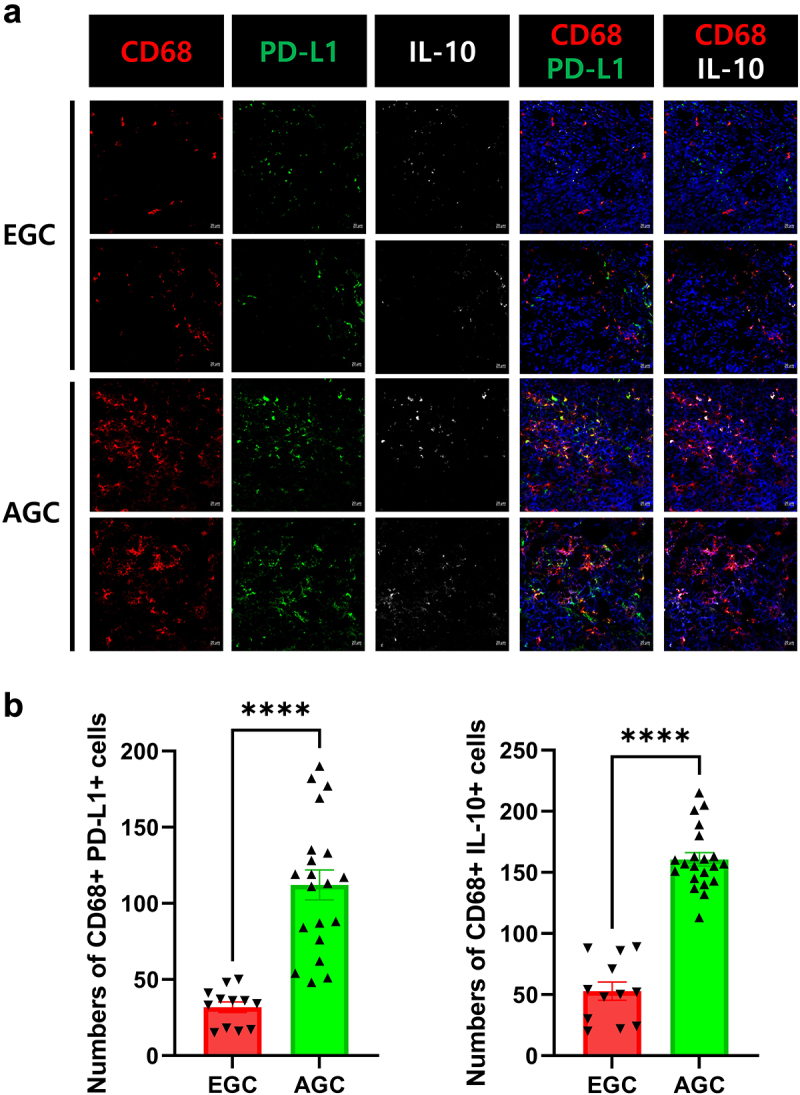
Increased levels of immunosuppressive markers in the tumor mucosa of AGC patients.

It is well known increased expression of immunosuppressive markers in various cancer type, including gastric cancer. We investigated the expression levels of immunosuppressive markers, including PD-L1 and IL-10, in the PBMCs (macrophages and dendritic cells) and cancer mucosa of GC patients. There were greater numbers of PD-L1- and IL-10-expressing macrophages (CD68^+^ cells) and PD-L1- and IL-10-expressing dendritic cells (CD11c^+^ cells), as well as PD-L1- and IL-10-expressing macrophages in the PBMC (Figure 1) and cancer mucosa (Figure 2), in AGC patients than in EGC patients. Our data indicated that immunosuppression is associated with GC progression. The clinicopathological characteristics of the participants are shown in Table 1.

PBMCs were isolated from HC, EGC, and AGC patients, and stimulated with 25 ng/mL PMA and 250 ng/mL ionomycin for 4 h. After stimulation, cells were stained with CD68, CD11c, PD-L1, and IL-10 antibodies and analyzed by flow cytometry. (**a**) Representative FACS plots show the population of CD68+PD-L1+, CD68+IL-10+, CD11c+PD-L1+, and CD11c+IL-10+. (**b**) Bar graphs showing mean percentages of CD68+PD-L1+ (left) and CD68+IL-10+ (right) cells in HC, EGC (*n* = 11) and AGC (*n* = 29) patients. (**c**) Bar graphs showing mean percentages of CD11c+PD-L1+ (left) and CD11c+IL-10+ (right) cells in EGC (*n* = 11) and AGC (*n* = 29) patients. Data are mean ± SD (*p < .05, **p < .01, ***p < .001).

Isolated tumor mucosa tissues were stained with CD68, CD11c, PD-L1, and IL-10 antibodies during surgery and analyzed using confocal microscopy. (a) Representative images show CD68+PD-L1+ and IL-10+ cells in the tumor mucosa of EGC (top) and AGC (bottom) patients. (b) Bar graphs showing the mean number of CD68+PD-L1+ (left, *n* = 3) and CD68+ IL-10+ (right, *n* = 5) cells in the tumor mucosa of EGC and AGC patients. Data are mean ± SD (****p < .001).

**Table 1.** Clinicopathological characteristics of the patients with early gastric cancer and advanced gastric cancer.

		Patient with EGC	Patient with AGC	P value
		N = 14	N = 34	
Age, mean (SD)		59.5(±12.61)	62.8235(±10.65)	<0.0001
Sex (M:F ratio)	M	10(71.4)	22(64.71)	0.340114
	F	4(28.6)	12(35.29)	
BMI, mean (SD)		23.92(±5.369)	24.34(±3.699)	<0.0001
ECOG	0	12(85.71)	27(79.41)	0.050037
	1	2(14.29)	6(17.65)	
	2	0	1(2.94)	
Smoking (%)	Never	3(21.42)	16(47.06)	0.011447
	Quit	7(50)	7(20.59)	
	Active	4(28.57)	11(32.35)	
Alcohol (%)	Never	8(57.14)	23(67.65)	0.167044
	Social	5(35.71)	8(23.53)	
	Heavy	1(7.14)	3(8.82)	
H. pylori infection	Don’t know	9	20	
	Negative	3	11	
	Positive	2	3	
Comorbidities	Non	4(28.57)	11(32.35)	0.762884
	Hypertension	5(35.71)	13(38.24)	
	DM	1(7.14)	2(5.88)	
	Cardiovascular	1(7.14)	1(2.94)	
	Pulmonary	1(7.14)	2(5.88)	
	others	2(14.29)	2(5.88)	
pT stage	T1	14		
	T2–4	0	34	
pN stage	N0	14	6(17.65)	0.050044
	N1–3	0	28(82.35)	
pM stage	M0	14	30(88.24)	0.001523
	M1	0	4(11.76)	
pStage 7^th^	I	14	7(20.59)	0.00023
	II	0	10(29.41)	
	III	0	13(38.24)	
	IV	0	4(11.76)	
Differentiation	Differentiated	5(35.71)	10(29.41)	0.197
	Undifferentiated	9(64.29)	24(70.59)	
Lauren	Intestinal	6(42.86)	8(23.53)	0.08618
	Diffuse/Mixed	8(57.14)	22(64.71)	
	Indeterminated	0	4(11.76)	
Lymphatic invasion	Negative	14	10(29.41)	0.012211
	Positive	0	24(70.59)	
Vascular invasion	Negative	14	28(82.35)	0.00645
	Positive	0	6(17.65)	
Neural invasion	Negative	14	16(47.06)	0.015401
	Positive	0	18(52.94)	

Data are numbers (percentages) or means (± SD). The chi-squared test was used to test for between-group differences in categorical variables; *P* < .05 was deemed indicative of statistical significance. BMI; body mass index; ECOG, Eastern Cooperative Oncology Group score.

### Dysbiosis in the gut microbiome of GC patients

**Figure 3.**
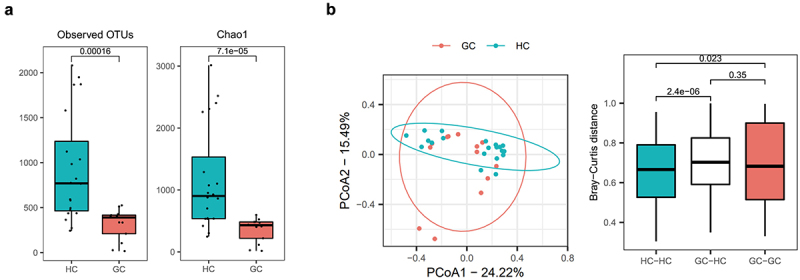
Gut microbiota profile of patients with GC.

**Figure 4.**
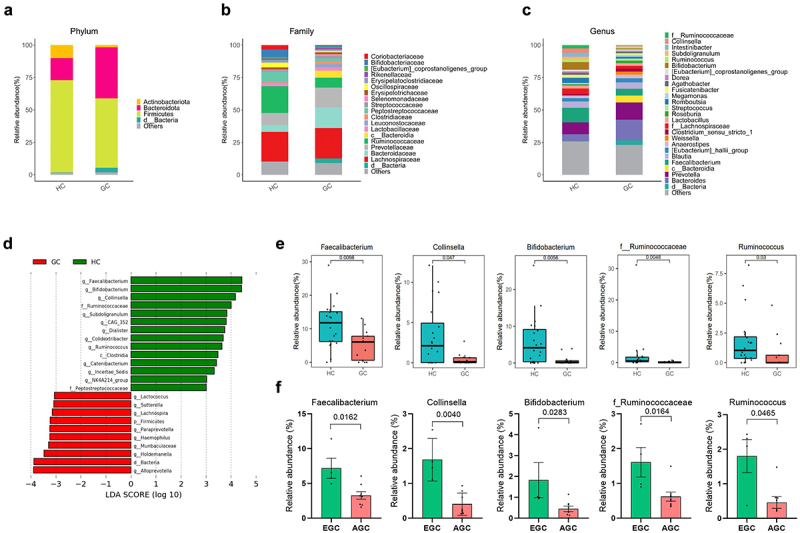
Differential microbial abundance in GC patients.

To investigate whether GC is associated with changes in the gut microbiome, stool samples from 20 HCs and 12 GC patients were sequenced. Compared to HCs, GC patients had a lower number of species and richness of the microbiome flora (Figure 3a). We evaluated and compared the overall diversity in gut microbiome composition between GC patients and HC using principal coordinate analysis reliant on Bray-Curtis dissimilarity (beta diversity). There was a significant difference in the intestinal flora of HCs and GC patients (Figure 3b). The gut microbial composition was altered in GC patients (Figure 4). At the phylum level, GC patients had lower abundances of *Actinomycetota* and *Bacillota*, and a higher abundance of *Bacteroidota*, compared to HCs (Figure 4a). At the family level, Bifidobacteriaceae and Ruminococcaceae, which are related to butyrate production, had lower abundances, whereas Bacteroidaceae had a higher abundance, in GC patients than in HCs (Figure 4b). At the genus level, there was also a reduction in butyrate producing bacteria within the intestinal flora of GC patients (Figure 4c). There were also remarkable differences at the genus level with distinct relative abundances of bacterial taxa between GC patients and HC linear discriminant analysis (LDA) Scores (Figure 4d). The abundances of *Faecalibacterium*, *Collinsella*, *Bifidobacterium*, f_ *Ruminococcus*, and *Ruminococcus*, i.e., butyrate-producing strains, were significantly reduced in GC patients compared to HCs (Figure 4e). Also, the abundances of *Faecalibacterium*, *Collinsella*, *Bifidobacterium*, f_ *Ruminococcus*, and *Ruminococcus*, i.e., butyrate-producing strains, were significantly reduced in AGC patients compared to EGC (Figure 4f).

Fecal samples were collected from HCs and GC patients, and analyzed for the gut microbiome. (a) Bar graphs showing observed OTUs (left) and Chao1 diversity (right). (b) Representative plots show principal coordinates analysis (left) and Bray-Curtis distance (right) between HCs and GC patients.

Gut microbiome of HCs and GC patients were analyzed at the phylum (a), family (b), and genus (c) levels. (d) Histogram showing the linear discriminant analysis (LDA) scores for bacterial abundances in HCs and GC patients. (e) Bar graphs showing relative abundances of *Faecalibacterium*, *Collinsella*, *Bifidobacterium*, f_ *Ruminococcus*, and *Ruminococcus* in HC and GC. (f) Bar graphs showing relative abundances of *Faecalibacterium*, *Collinsella*, *Bifidobacterium*, f_ *Ruminococcus*, and *Ruminococcus* in EGC and AGC.

### Butyrate treatment inhibits PD-L1 and IL-10 expression in the PBMCs of GC patients

**Figure 5.**
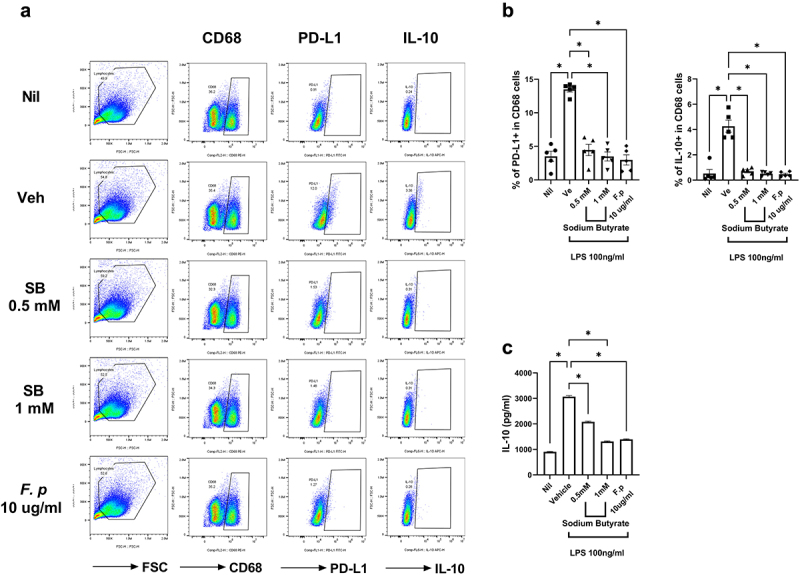
Reduction of PD-L1 and IL-10 levels by butyrate.

The butyrate-producing strains, including *Faecalibacterium*, had lower abundances in GC patients than in HCs. We investigated the roles of butyrate and *Faecalibacterium* in GC patients. The administration of butyrate and *Faecalibacterium* into the PBMCs of GC patients and THP-1 cells decreased the number of PD-L1- and IL-10-expressing macrophages (Figure 5a, b and Supplementary Figure S1). In addition, administration of butyrate and *Faecalibacterium* decreased the concentration of IL-10 (Figure 5c).

Isolated PBMCs from patients with GC were cultured with 100 ng/mL LPS in the absence or presence of 0.5 mM of butyrate or 10 μg/mL *Faecalibacterium* for 72 h. After 72 h, the supernatant was harvested for ELISA and cells were stimulated with 25 ng/mL PMA and 250 ng/mL ionomycin for 4 h. After stimulation, cells were stained with antibodies against CD68, PD-L1, and IL-10 for flow cytometry. (a) Bar graphs showing mean percentages of PD-L1 (left) and IL-10 (right) of CD68^+^ cells in the indicated conditions. (b) Bar graph showing the mean IL-10 level in the supernatant of the indicated conditions. (c) Bar graphs showing the mean percentage of PD-L1 (left) and IL-10 (right) of CD68^+^ cells in the indicated conditions. Data are mean ± SEM (*p < .05, **p < .01, ***p < .001).

### Butyrate suppresses the growth of GC cells

**Figure 6.**
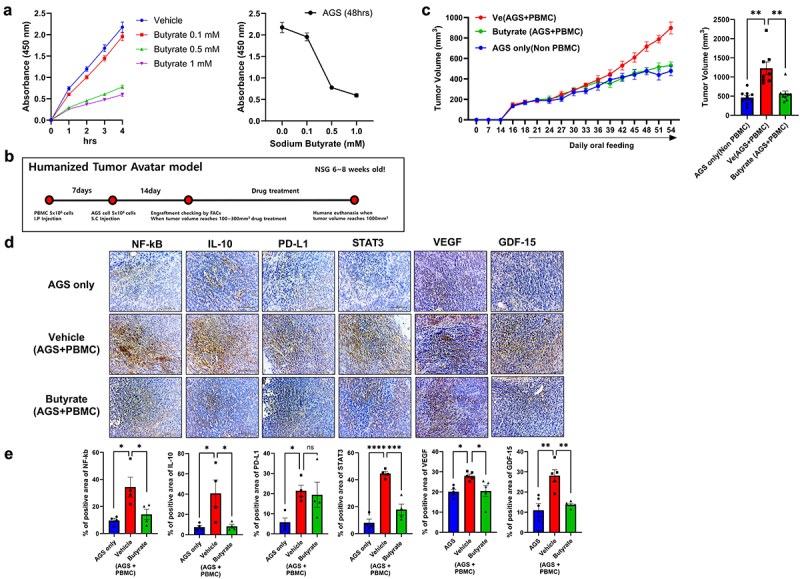
Inhibition of tumor cell growth by butyrate.

Next, we investigated the effects of butyrate on the growth of GC cells. We cultured AGS cells, a gastric cancer cell line, with butyrate and measured the cell growth. Butyrate significantly suppressed the growth of AGS cells in an *in vitro* culture system (Figure 6a). Next, we investigated the effect of butyrate on GC cell growth *in vivo*. For this, we subcutaneously injected a humanized tumor mouse model with AGS cells and PBMCs from healthy control or GC patients in the absence or presence of butyrate (Figure 6b, c and Supplementary Figure S2). Butyrate administration significantly decreased the tumor size and levels of the immunosuppressive markers (PD-L1 and IL-10), their regulators (NF-_K_B and STAT3), and cancer growth factor (VEGF and GDF-15) in cancer tissues (Figure 6d, e). Our data show the potential of butyrate as a therapeutic utility via suppressing cancer cell growth in gastric cancer.

(a) AGS cells were cultured with different concentration of butyrate for 48 h. Cell growth was measured using CCK8 cell counting kit. (b) 5 × 10^6^ PBMCs from GC patients were injected into NSG mice. Seven days after PBMC injection, 5 × 10^6^ AGS cells were subcutaneously injected into mice. Fourteen days after the injection of AGS cells, blood samples were collected for flow cytometry. Then, 200 mg/kg of butyrate was administered orally to mice every day. The mice were euthanized at 54 days after the experiment initiation. (c) Graphs (top) showing tumor size in the Veh (PBMCs only), butyrate (PBMCs, AGS cells, and butyrate), and AGS (AGS only) groups. Bar graph (bottom) showing mean tumor size in the indicated group. (d) NF-_K_B, IL-10, PD-L1, STAT3, VEGF, and GDF-15 expression levels in the tumor tissues of the indicated groups were assessed using immunohistochemistry. (e) Bar graphs showing the mean percentages of NF-_K_B-, IL-10-, PD-L1-, and STAT3-positive area. Data are mean ± SEM (*p < .05, **p < .01).

## Discussion

Several cancer types, including GC, are associated with immunosuppression.^26,27^ Several studies have reported an association between the levels of immunosuppressive markers and GC progression.^28–30^ In the present study, we evaluated the levels of immunosuppressive markers (PD-L1 and IL-10) in the peripheral blood and tumor tissues of GC patients. The levels of markers in the peripheral blood and tumor tissues were significantly higher in AGC patients than in EGC patients.

Microorganisms are abundant in the intestinal system and oral cavity.^31^ Reduced microbial diversity predisposes one to various diseases, such as cancer, rheumatoid arthritis, Crohn’s disease, obesity, and diabetes.^32–34^ Previous studies have found that the gut microbiome is an etiological factor in GC.^35–38^ GC is associated with reduced microbial diversity.^36,39^ Sarhadi *et al*.^40^ reported reduced microbial diversity and richness in gastrointestinal stromal tumors as well as differences in gut microbiota according to the GC type (e.g., diffuse, gastrointestinal stromal tumors, intestinal, and mixed).

Several recent studies have found that the microbiome and its metabolites, such as SCFAs, have antitumor effects and are associated with enhanced treatment responses to immunotherapy. Dikeocha *et al*.^41^ found that *Faecalibacterium* inhibits tumorigenesis in colorectal cancer and suppresses proliferation of colorectal cancer cells. In addition, *Bifidobacterium* and *Lactobacillus* inhibit GC cell growth.^42^ Recent studies have shown that the SCFA butyrate enhances the therapeutic effects of chemotherapy^43^ and radiotherapy.^44^

Recently, Liang *et al*. evaluated the anti-tumor effect of butyrate *in vitro* and *in vivo* systems.^45^ They found that butyrate inhibits proliferation, migration, and invasion of tumor cells. Furthermore, butyrate inhibited aerobic glycolysis in tumor cells via inhibiting wnt/β-catenin/c-Myc signaling pathway. However, no previous studies have evaluated the antitumor effects of butyrate in humanized model which mimics pathophysiological condition of human gastric cancer. In this study, we evaluated the anti-tumor effect of butyrate in humanized mouse model.

We found gut dysbiosis in GC patients. The abundances of the butyrate-producing bacteria *Faecalibacterium*, *Collinsella*, *Bifidobacterium*, *f_Ruminococcaceae*, and *Ruminococcus*^46,47^ were decreased in GC patients. We hypothesized that reduced butyrate level due to decreased abundances of these taxa was associated with immunosuppression. In line with this, butyrate treatment reversed the immunosuppression in GC. To test our hypothesis, we explored the effects of butyrate *in vitro* and *in vivo*. In the *in vitro* experiment, butyrate treatment reduced the expression levels of PD-L1 and IL-10 in CD68-positive PBMCs of GC patients and inhibited the growth of GC cells. To test the effect of butyrate *in vivo*, we designed a humanized tumor mouse model and found reduced tumor growth with butyrate treatment. In addition, the expression levels of IL-10 and PD-L1 were decreased in the butyrate-treated group. Interestingly, butyrate treatment decreased the expression levels of NF-kB and STAT3. A recent study showed that STAT3 and NF-kB increased the expression level of PD-L1 in cancer.^48,49^ Our results showed that butyrate regulates the expression levels of immunosuppressive markers, particularly PD-L1, through the modulation of STAT3 and NF-kB expression. Vascular endothelial growth factor (VEGF) and growth differentiation factor 15 (GDF15) are known to promote tumor growth through coordinating angiogenesis and participating tumor invasion and their expression was enhanced in tumor patients.^50,51^ It has been reported that the expression of VEGF is inhibited by IFN-γ.^52^ We found increased expression of VEGF and GDF15 in the group of injection of tumor tissue with patient PBMC which produce less IFN-γ. Our data suggest that increased VEGF and GDF15 lead promoting tumor growth via immune suppression of patient PBMC.

Humanized mouse models are commonly used to explore the mechanisms of human diseases. We have established humanized mouse models for several diseases, including systemic sclerosis^53^ and liver transplant.^54^ In the present study, we established a humanized GC mouse model to explore the role of immune cells in GC, which have immunosuppressive functions. The administration of PBMCs from GC patients enhanced tumor cell growth, whereas butyrate inhibited tumor cell growth by inhibiting the expression of immunosuppressive markers, including PD-L1 and IL-10. Our results suggest that regulation of immunoactivity is a key target for cancer treatment and affects the prognosis of GC.

Our study had a few limitations. First, we used human PBMCs to generate our mouse model, which requires 5 × 10^6^ PBMCs per animal. Because of the small number of cells, we could not generate multiple animals for a single experiment. Second, the cancer cells used in the present study may have led to inaccurate results. In the PDX model, cancer tissues from patients are transplanted, whereas we transplanted AGS cells, a GC cell line, via subcutaneous injections. There may be discrepancies in the results obtained from cancer tissues obtained from patients and cancer cell lines. In the humanized mouse model, certain cell types cannot be engrafted. Future studies should engraft cancer cells from GC patients into the mouse model.

Despite our study limitations, our results provide insight into the relationships between the microbiome and its metabolites and immunosuppression. This study was the first to identify an antitumor effect of butyrate, a representative metabolite, in GC via the regulation of the expression of immunosuppressive markers. Our findings suggest that butyrate has therapeutic effects in GC via the regulation of immunosuppressive markers.

## Supplementary Material

Lee et al_Supplementary Information clean.docx

## Data Availability

The authors confirm that the data supporting the findings of this study are available within the article. Gut microbiome analysis was deposited in the BioProject and Sequence Read Archive (SRA; https://www.ncbi.nlm.nih.gov/sra) under the accession numbers PRJNA937852.
