# Supplementary material for: Gut microbiome-derived butyrate inhibits the immunosuppressive factors PD-L1 and IL-10 in tumor-associated macrophages in gastric cancer
Source: Gut Microbes. 2024 Jan 10;16(1):2300846. doi: 10.1080/19490976.2023.2300846 (PMC10793689; doi:10.1080/19490976.2023.2300846)

**Original article**

**Gut microbiome-derived butyrate inhibits the immunosuppressive factors PD-L1 and IL-10 in tumor-associated macrophages in gastric cancer**

Seung Yoon Lee^1,2,3^, JooYeon Jhun^1,2,3^, Jin Seok Woo^1,2^, Kun Hee Lee^1,2,3^, Sun-Hee Hwang^1,2^, Jeonghyeon Moon^4^, Goeun Park^5^, Sun Shim Choi^5^, So Jung Kim^6^, Yoon Ju Jung^7^, Kyo Young Song^6,#^, Mi-La Cho^1,2,3,8, #^

^1^ Rheumatism Research Center, Catholic Research Institute of Medical Science, The Catholic University of Korea, Seoul 06591, Korea

^2^ Lab of Translational ImmunoMedicine, Catholic Research Institute of Medical Science, College of Medicine, The Catholic University of Korea, Seoul 06591, Korea

^3^ Department of Biomedicine & Health Sciences, College of Medicine, The Catholic University of Korea, Seoul 06591, Korea

^4^ Departments of Immunobiology and Neurology, Yale School of Medicine, New Haven, CT 06520, USA

^5^ Division of Biomedical Convergence, College of Biomedical Science, Institute of Bioscience & Biotechnology, Kangwon National University, Chuncheon, 24341, Korea

^6^ Division of Gastrointestinal Surgery, Department of Surgery, Seoul St. Mary’s Hospital, College of Medicine, The Catholic University of Korea, Seoul 06591, Korea

^7^ Division of Gastrointestinal Surgery, Department of Surgery, Yeouido St. Mary’s Hospital, Seoul 07345, Korea

^8^ Department of Medical Life Sciences, College of Medicine, The Catholic University of Korea, Seoul 06591, Korea

^#^ Authors to whom correspondence should be addressed.

**Kyo Young Song, M.D., Ph.D.**, Department of Surgery, Seoul St. Mary’s Hospital, College of Medicine, The Catholic University of Korea, Seoul 06591, Korea (Tel: +82-2-2258-6106; Fax: +82-2-595-2876; E-mail: [skys9615@gmail.com](mailto:skys9615@gmail.com) )

**Mi-La Cho, Ph.D.**, Rheumatism Research Center, Catholic Research Institute of Medical Science, College of Medicine, The Catholic University of Korea, Seoul 06591, Korea (Tel: +82-2-3147-8832; Fax: +82-2-2258-7473; E-mail: [iammila@catholic.ac.kr](mailto:iammila@catholic.ac.kr))**Supplementary Figure 1. Reduction of PD-L1 and IL-10 levels by butyrate.**

THP-1 cells differentiated with 150 ng/mL PMA for 24 hr. After that, cells were cultured with 0.5 mM or 1 mM butyrate for 72 hr and stimulated with 25 ng/mL PMA and 250 ng/mL ionomycin for 4 h. After stimulation, cells were stained with antibodies against CD68, PD-L1, and IL-10 for flow cytometry. **A**. Representative facs plot show the population of CD68^+^PD-L1^+^ and CD68^+^IL-10^+^. **B**. Bar graphs show average % of CD68^+^PD-L1^+^ and CD68^+^IL-10^+^ population.


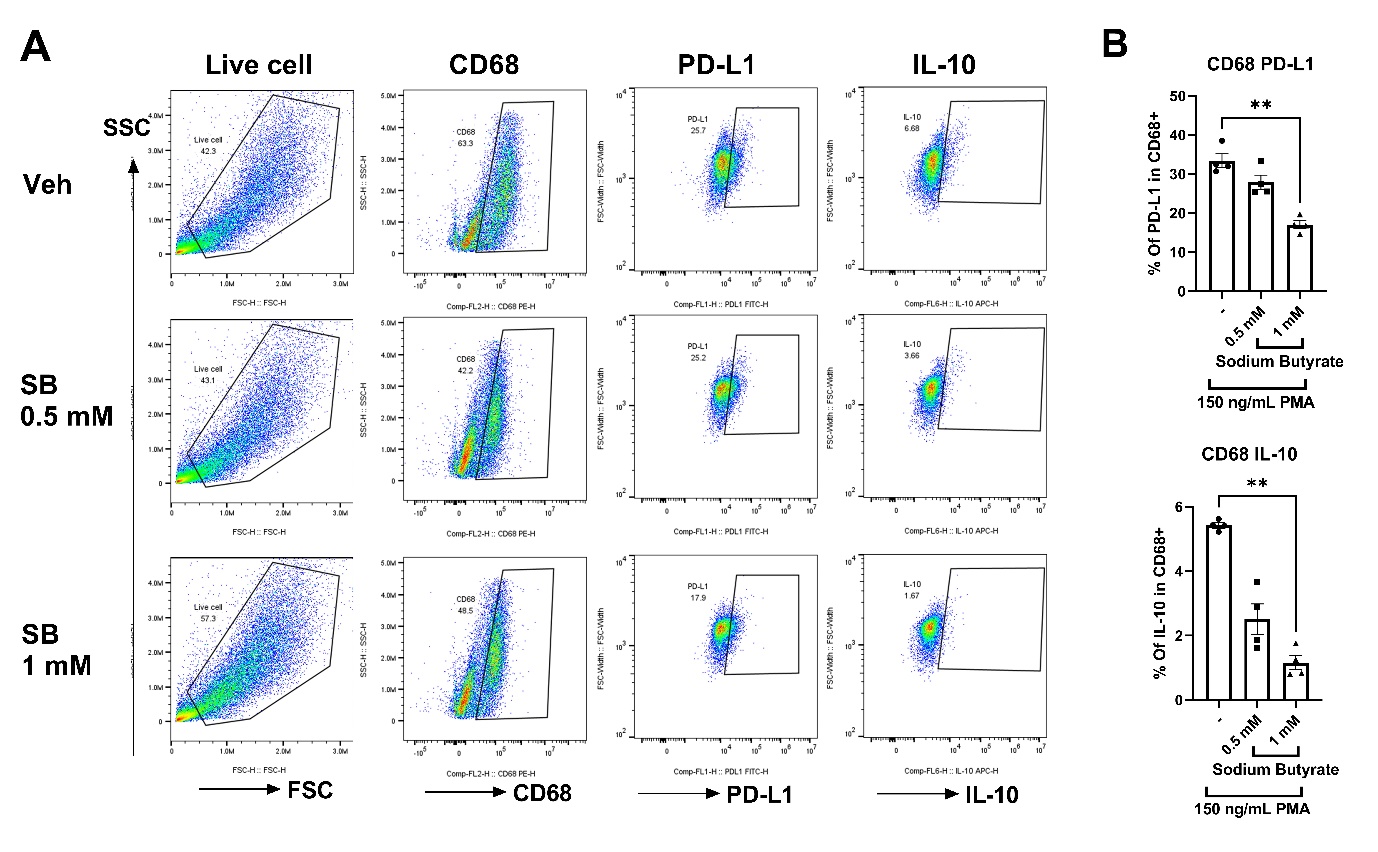


**Supplementary Figure 2. The effect of PBMC from healthy control or gastric cancer patients in tumor growth. A.** 5 × 10^6^ PBMCs from GC patients were injected into NSG mice. Seven days after PBMC injection, 5 × 10^6^ AGS cells were subcutaneously injected into mice. Fourteen days after the injection of AGS cells, blood samples were collected for flow cytometry. The mice were euthanized at 48 days after the experiment initiation. Tumor size was evaluated every 3 days. **B**. 5 × 10^6^ PBMCs from healthy control or GC patients were injected into NSG mice. Seven days after PBMC injection, 5 × 10^6^ AGS cells were subcutaneously injected into mice. Fourteen days after the injection of AGS cells, blood samples were collected for flow cytometry. The mice were euthanized at 51 days after the experiment initiation. Tumor size was evaluated every 3 days.


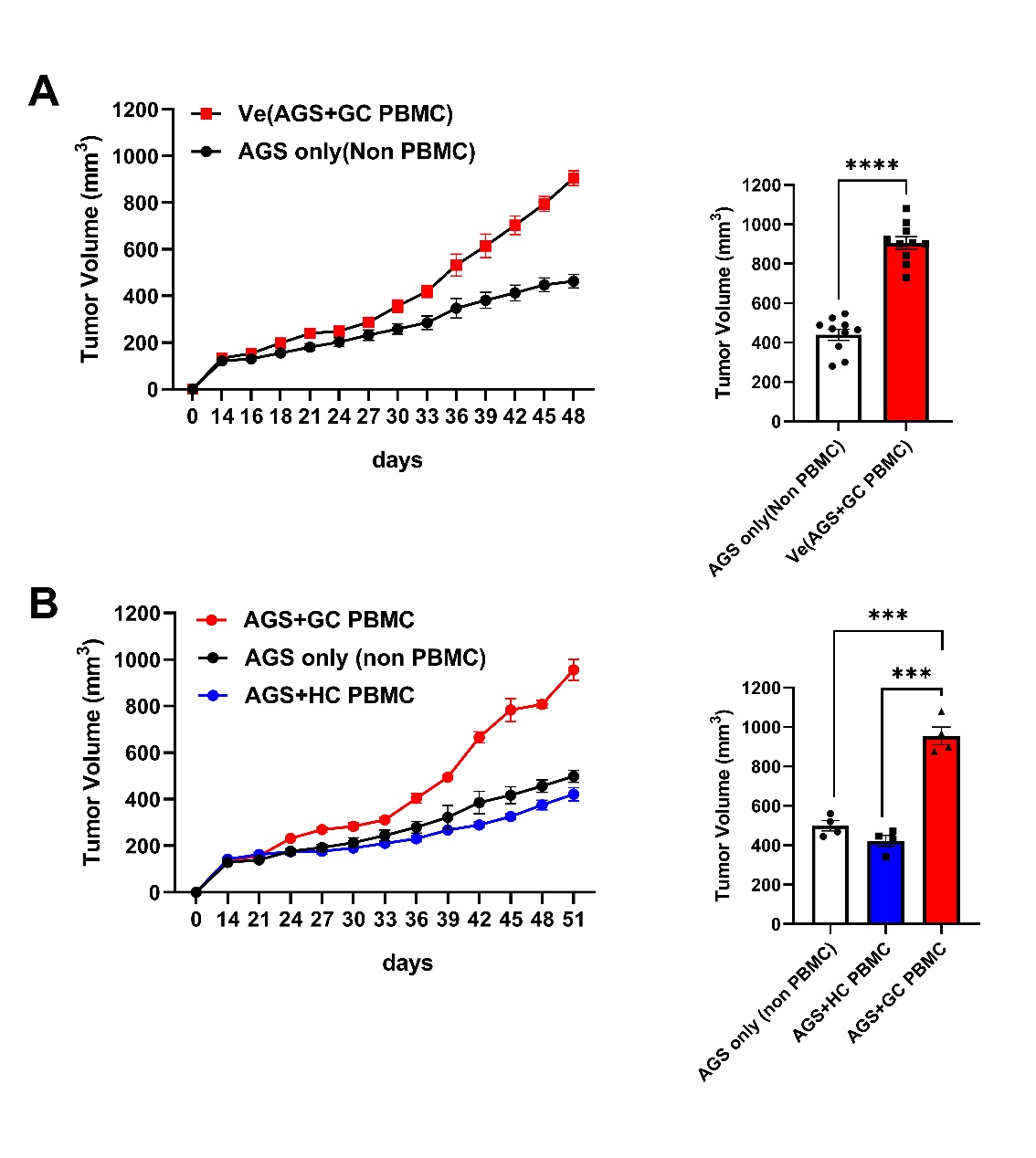

Supplement: Lee et al_Supplementary Information clean.docx [file KGMI_A_2300846_SM8743.docx]
